# Mental State Recognition Deficits Linked to Brain Changes in Parkinson's Disease Without Dementia

**DOI:** 10.1111/ejn.70014

**Published:** 2025-02-17

**Authors:** Giulia Funghi, Giuseppe Rabini, Claudia Meli, Chiara Speranza, Enrica Pierotti, Francesca Saviola, Stefano Tambalo, Francesca Zappini, Giorgio Fumagalli, Luca Turella, Jorge Jovicich, Costanza Papagno, Alessandra Dodich

**Affiliations:** ^1^ Center for Mind/Brain Sciences (CIMeC) University of Trento Rovereto Italy; ^2^ Ecole Polytechnique Federale de Lausanne, Medical Image Processing Lab (MIP: Lab) Neuro‐X Institute Lausanne Switzerland; ^3^ Department of Radiology and Medical Informatics University of Geneva Geneva Switzerland

**Keywords:** emotion, face processing, functional MRI, magnetic resonance imaging

## Abstract

Recent studies have reported social cognitive deficits, particularly in emotional processing, in Parkinson's disease (PD). However, a comprehensive characterization of these deficits and their underlying neural correlates remains elusive. Therefore, this study aims to investigate the association between deficits in the recognition of complex mental states and structural/functional brain changes in non‐demented PD individuals. To reach this aim, 24 PD participants underwent clinical assessment, neuropsychological testing and the *FAcial Complex Expressions* (FACE) test, a novel test of complex mental state recognition from faces. Patients were classified as clinically impaired (*n* = 8) or unimpaired (*n* = 16) based on performance on this test. Magnetic resonance imaging data were acquired to investigate the association between FACE test performance and both resting‐state functional connectivity and grey matter volume, within the emotion understanding network and at the whole‐brain level. Statistical analyses also included the comparison of imaging metrics between the impaired and unimpaired groups. Results showed that complex mental state recognition in PD was significantly associated with both defective and compensatory mechanisms at the functional and anatomical level within the emotion understanding network, particularly involving the amygdala, dorsomedial prefrontal cortex, primary/secondary somatosensory cortices, and right anterior temporal cortex. Whole‐brain results extended the network to temporal and medial frontal areas. In conclusion, reduced recognition of complex mental states in non‐demented PD patients is associated with alterations in the emotion understanding network A comprehensive characterization of early emotional deficits in these patients may have significant implications in the characterization of the cognitive phenotype, with potential benefit for tailored non‐pharmacological intervention.

## Graphical abstract

Functional and structural changes in the emotion processing neural network have been observed in Parkinson's disease patients. Results show that impaired mental state recognition in PD is associated with early amygdala dysfunction, as well as alterations in regions involved in emotional cue detection (i.e., the amygdala), emotion categorization and maintenance of semantic knowledge about the social world. Whole brain analyses extended the complex mental state recognition network to frontal and temporal areas.

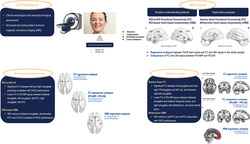

**Abbreviations**
ACC: anterior cingulate cortex
Amy: amygdala
ATC: anterior temporal cortex
CSF: cerebrospinal fluid
dACC: dorsal anterior cingulate cortex
dlPFC: dorsolateral prefrontal cortex
dmPFC: dorsomedial prefrontal cortex
dTL: dorsal portion of the temporal lobe
EPI: echo‐planar imaging
FACE: facial complex expressions test
FC: functional connectivity
fMRI: functional magnetic resonance imaging
GM: grey matter
ILFC: inferior lateral frontal cortex
Ins: insula
LEDD: levodopa equivalent daily dose
MoCA: Montreal Cognitive Assessment test
MRI: magnetic resonance imaging
OFC: orbitofrontal cortex
PD: Parkinson's disease
PD‐IMP: PD patients classified as impaired on the FACE test performance according to Italian normative data
PD‐UN: PD patients classified as unimpaired on the FACE test performance according to Italian normative data
PFC: prefrontal cortex
pSTS: posterior part of the superior temporal sulcus
TE: echo time
TR: repetition time
RRC: ROI‐to‐ROI connectivity
rs‐fMRI: resting state‐functional MRI
S1/S2: primary and secondary somatosensory cortices
SBC: seed‐based connectivity maps
TIV: total intracranial volume
ToM: theory of mind
vACC: ventral anterior cingulate cortex
VBM: voxel‐based morphometry
vlPFC: ventrolateral prefrontal cortex
vmPFC: ventromedial prefrontal cortex
vTL: ventral portion of the temporal lobe
WM: white matter

## 1 Introduction

Parkinson's disease (PD) is a progressive multi‐system neurodegenerative disorder characterized by progressive dopamine depletion in the basal ganglia, frontoparietal regions and limbic system (Obeso et al. 2008). These brain systems, responsible for motor, cognitive and affective functions, also play a relevant role in social cognition, a complex and multifaceted cognitive domain involving multiple processes that collectively underlie everyday social interactions (Happé, Cook, and Bird 2017).

Although initially under‐reported, in recent years numerous reviews and meta‐analyses have consistently provided evidence of social‐cognitive dysfunctions in PD patients, particularly in emotional processing (Argaud et al. 2018; Gray and Tickle‐Degnen 2010; Palmeri et al. 2017; Péron et al. 2012), theory of mind (Bodden, Dodel, and Kalbe 2010; Bora, Walterfang, and Velakoulis 2015; Coundouris, Adams, and Henry 2020; Palmeri et al. 2017), and empathy (Coundouris, Adams, and Henry 2020), with significant difficulties in both emotion recognition and representation (Enrici et al. 2015). Such deficits can drastically reduce the quality of life of PD patients and families (Schwartz et al. 2020), possibly increasing demands on the health system.

Recently, our research group demonstrated the presence, detectable at clinical assessment, of significant deficits in the recognition of complex mental states from faces in a relevant proportion of PD patients (28%) (Terruzzi et al. 2023); however, despite extensive theoretical and behavioural research, the characterization of these affective deficits in terms of underlying neural correlates remains still strongly limited (Baggio et al. 2012; Diederich et al. 2016; Lewis and Ricciardi 2021; Rabini et al. 2023; Trompeta, Fernández Rodríguez, and Gasca‐Salas 2021; Wabnegger et al. 2015), with the majority of studies focusing on basic emotion recognition and its structural neural substrates (Baggio et al. 2012; Ibarretxe‐Bilbao et al. 2009).

Spunt and Adolphs (Spunt and Adolphs 2019) recently proposed a model of emotion understanding which outlines the cognitive processes involved and synthetizes the available (mainly functional) neuroimaging data on brain regions associated with emotion understanding. According to this model, the first step is represented by all the subprocesses required for the *detection and orientation of attention* to emotion‐relevant sensory cues in the environment, which are associated with amygdalar function. The recruitment of temporal regions (i.e., the anterior temporal cortex and the posterior superior temporal sulcus) enables the *categorization* of these cues in emotional terms. Regions such as the primary and secondary somatosensory cortex as well as the insula contribute to this process via the *embodied simulation* of specific emotions. Finally, regions belonging to the frontal lobe (i.e., the dorsomedial prefrontal cortex and the ventrolateral prefrontal cortex) are recruited for the *causal attribution* of the affective signals.

Overall, preliminary evidence in PD patients indicates the presence of functional and structural changes in these regions, which have been related to reduced performance in basic emotion recognition (Baggio et al. 2012; Benzagmout et al. 2019; Ibarretxe‐Bilbao et al. 2009; Suzuki et al. 2006; Tessitore et al. 2002; Wabnegger et al. 2015). In particular, available studies investigating the neural correlates of emotion recognition impairment in PD have found an association between reduced abilities and structural neurodegeneration in limbic regions, including the orbitofrontal cortex, the amygdala, the anterior cingulate cortex, the insula, and the ventral striatum (Baggio et al. 2012; Diederich et al. 2016; Ibarretxe‐Bilbao et al. 2009; Suzuki et al. 2006). Conversely, evidence for functional changes in PD associated with deficits in basic emotion recognition is more limited, with only a few studies supporting an association with functional alterations in limbic (Bell et al. 2019; Tessitore et al. 2002) and parietal (Wabnegger et al. 2015) regions. The collective findings suggest that the potential for deficits in emotion processing may be associated with alterations in regions belonging to the ‘social brain’ (Frith 2007) in PD patients. However, evidence for this hypothesis is still severely limited to the recognition of basic emotions and, to the best of our knowledge, no studies have investigated the neural correlates of reduced recognition of complex mental states in this population from both a structural and functional perspective. The aim of the current study is therefore to extend the characterization of these deficits in non‐demented PD patients, by investigating the association between altered recognition of complex mental states from faces and brain changes using a multi‐level neuroimaging approach (i.e., functional and structural).

Specifically, the first aim of this study is to investigate the relationship between performance on a test of complex mental state recognition, the *FAcial Complex Expressions* (FACE) test (Terruzzi et al. 2023), and both resting‐state functional connectivity (FC) and grey matter (GM) volume, taking into account the brain areas involved in the emotion understanding networks via region of interest (ROI) analysis (Spunt and Adolphs 2019). A second exploratory aim is to investigate the association between FACE performance and functional/structural brain metrics at the whole‐brain level, as this may potentially extend the network of regions involved in emotion understanding in these patients.

## 2 Materials and Methods

### 2.1 Participants

Twenty‐four non‐demented patients with PD according to the United Kingdom Parkinson's Disease Society brain bank criteria (Hughes et al. 1992) (13 male; mean age: 67.1 ± 7.0 years; mean education: 10.8 ± 3.8 years; mean MOCA score: 22.4 ± 3.9) were recruited at the Centre for Neurocognitive Rehabilitation of the Centre for Mind/Brain Sciences–CIMeC (University of Trento). All patients underwent a comprehensive clinical neurological assessment and a complete neuropsychological evaluation, including the Montreal Cognitive Assessment (MoCA test) as a screening test (Conti et al. 2015). Inclusion criteria were as follows: (I) a diagnosis of idiopathic PD; (II) a Hoehn and Yahr score ≤3 (Hoehn and Yahr 1967); (III) being under antiparkinsonian medication; and (IV) age over 50 years. Patients with dementia, based on the results of the comprehensive neuropsychological assessment and the impact of cognitive deficits on daily living activities (Emre et al. 2007), or a history of neuropsychiatric disorders (e.g., major depression, psychosis or substance abuse) were excluded from the study. No participants exhibited contraindications to magnetic resonance imaging (MRI) scanning based on medical history and physical examination. The study was conducted with patients taking their usual L‐dopa medication (ON state), which was quantified by conversion to Levodopa Equivalent Daily Dose (LEDD) (not available for four patients).

All participants provided informed consent for the study, which adhered to the ethical guidelines of the local ethics committee (University of Trento Research Ethics Committee, protocol 2019‐033) and the Declaration of Helsinki (World Medical Association 2013).

### 2.2 Mental State Recognition Task

**FIGURE 1:**
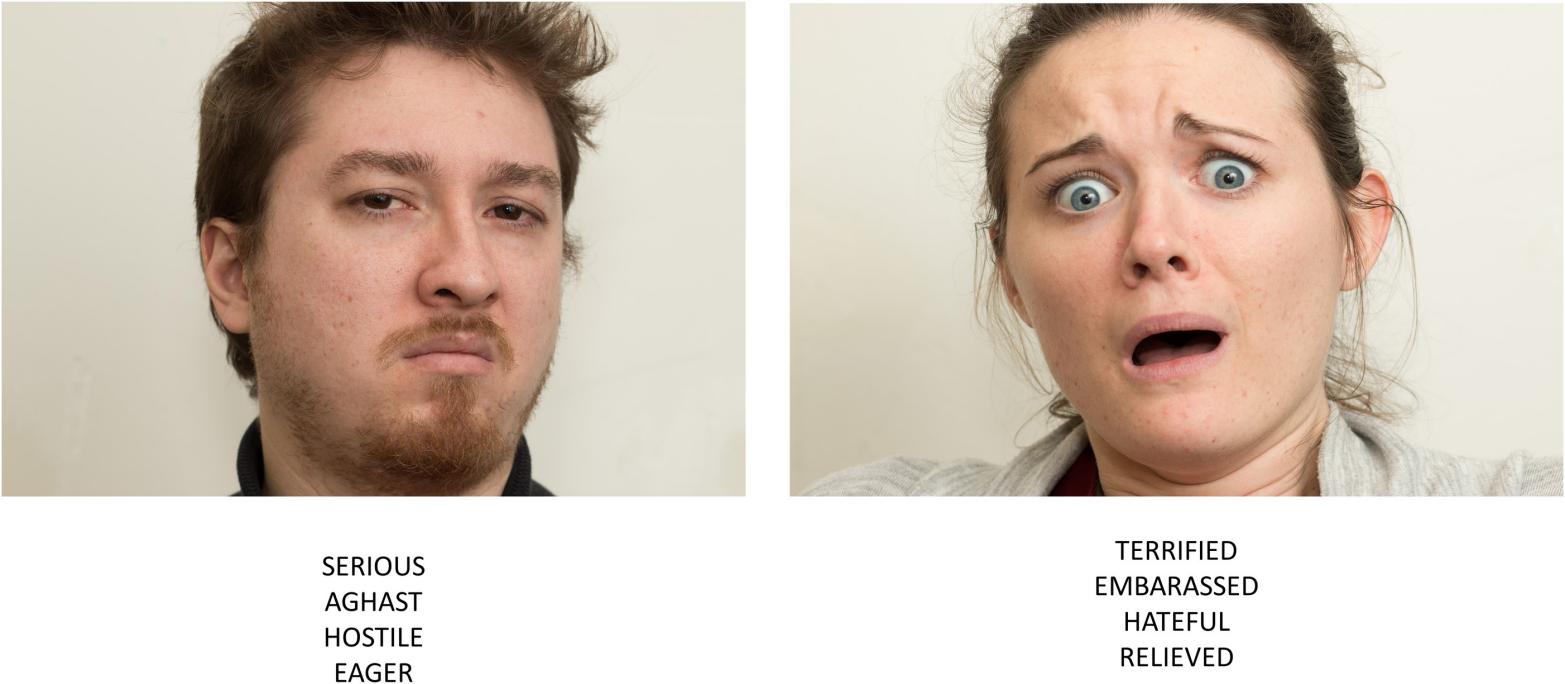
Examples of pictures from the FACE test. The figure shows two actors (male and female) displaying the facial expression corresponding to ‘Hostile’ and ‘Terrified’.

The ability to recognize complex mental states was assessed using the *Facial Complex Expressions (*FACE) test (Terruzzi et al. 2023), a novel task developed in Italy and validated in both healthy controls and patients with PD, showing satisfactory psychometric properties (Terruzzi et al. 2023). The test consists of 36 pictures of facial expressions representing different mental states and interpreted by professional actors. For each picture, participants were required to provide a verbal response by selecting from four adjectives the one that most accurately described the actor's facial expression. Illustrative examples of FACE pictures are presented in Figure 1.

### 2.3 MRI Data Acquisition and Processing

#### 2.3.1 MRI Acquisition Protocol

The MRI data were collected at the Centre for Mind/Brain Sciences ‐ CIMeC (University of Trento) using a 3.0 T MRI scanner (Prisma, Siemens) with a 64‐channel head receive coil. The procedure was adequately described to all PD subjects, who were instructed to keep their eyes closed and not to sleep during the MRI scan. Soft pads were placed around the patient's head to minimize head movement during the MRI scan. The MRI scan was performed within 3 months of the behavioural data collection. For each participant, T1‐weighted MEMPRAGE structural images were acquired using the following parameters: MPRAGE_GRAPPA: 176 volumes, isotropic voxel resolution of 1 mm, sagittal plane orientation, flip angle of 7°, matrix = 256 × 256, repetition time (TR) of 2.53 s, echo time (TE) of 1.35, 3.07, 4.79, 6.51 ms, TI of 1100 ms, slice thickness of 1 mm. Additionally, resting‐state functional magnetic resonance images (rs‐fMRI) were acquired using echo planar imaging (EPI) T2*‐weighted scans. The following acquisition parameters were used: TE of 28 ms, TR of 1.0 s, flip angle of 59°, axial slice thickness of 2 mm. A total of 400 whole brain volumes were acquired in a resting state run of 6 min 40 s, isotropic voxel size 2 mm, AC/PC aligned.

#### 2.3.2 Functional Connectivity Pre‐Processing

rs‐fMRI analyses were performed using CONN functional connectivity toolbox (release 21.a‐RRID: SCR_009550; https://web.conn‐toolbox.org; Nieto‐Castanon 2021; Whitfield‐Gabrieli and Nieto‐Castanon 2012), and Statistical Parametric Mapping (SPM12‐v7487‐RRID: SCR_007037; https://www.fil.ion.ucl.ac.uk/spm; Penny et al. 2011), based on MATLAB R2020b.

Functional and anatomical data were pre‐processed using a flexible pre‐processing pipeline, including (a) realignment with correction of susceptibility‐distortion interactions, (b) slice timing correction, (c) outlier detection, (d) direct segmentation and normalization to the Montreal Neurological Institute (MNI) space and (e) smoothing (only for voxel‐level analyses, not for ROI‐level analyses). Functional data were realigned using the SPM realign and unwarp procedure, where all scans were coregistered to a reference image (first scan of the first session) using a least squares approach and a six‐parameter (rigid body) transformation, and resampled using b‐spline interpolation to correct for motion and magnetic susceptibility interactions. Temporal misalignment between different slices of the functional data (acquired in interleaved Siemens order) was corrected according to the SPM slice‐timing correction (STC) procedure, using sinc temporal interpolation to resample each slice BOLD time‐series to a common mid‐acquisition time. Potential outlier scans were identified using ART as acquisitions with framewise displacement above 0.9 mm or global BOLD signal changes above 5 standard deviations, and a reference BOLD image was computed for each subject by averaging all scans excluding outliers. Functional and anatomical data were normalized into standard MNI space, segmented into grey matter (GM), white matter (WM) and cerebrospinal fluid (CSF) tissue classes, and resampled to 2‐mm isotropic voxels following a direct normalization procedure using SPM unified segmentation and normalization algorithm with the default IXI‐549 tissue probability map template. Last, functional data (only for voxel‐level analyses) were smoothed using spatial convolution with a Gaussian kernel of 8‐mm full width half maximum (FWHM).

In addition, functional data were denoised using a standard denoising pipeline including the regression of potential confounding effects characterized by white matter timeseries (five CompCor noise components), CSF timeseries (five CompCor noise components), motion parameters and their first order derivatives (12 factors), outlier scans (below 123 factors), session effects and their first order derivatives (2 factors) and linear trends (two factors) within each functional run, followed by bandpass frequency filtering of the BOLD timeseries between 0.008 Hz and 0.09 Hz. CompCor noise components within white matter and CSF were estimated by computing the average BOLD signal as well as the largest principal components orthogonal to the BOLD average, motion parameters and outlier scans within each subject's eroded segmentation masks. From the number of noise terms included in this denoising strategy, the effective degrees of freedom of the BOLD signal after denoising were estimated to range from 41.2 to 61.3 (average 58.8) across all subjects.

#### 2.3.3 Grey Matter Pre‐Processing

Anatomical images were analysed using the Computational Anatomy Toolbox (CAT12.8.2‐r2170; https://neuro‐jena.github.io/cat; Gaser et al. 2022) for Statistical Parametric Mapping (SPM12‐v7771; https://www.fil.ion.ucl.ac.uk/spm; Penny et al. 2011) in MATLAB R2019a.

The T1‐weighted anatomical images were pre‐processed according to the standard VBM pre‐processing pipeline of CAT 12, which includes: (a) tissue segmentation into GM, WM and CSF, (b) spatial normalization to the MNI space, (c) modulation and (d) spatial smoothing with a Gaussian kernel of 8‐mm full width at half maximum (FWHM) (Gaser et al. 2022).

In addition, to assess data quality, we visually inspected each segmented and modulated image and considered the Image Quality Ratings (IQR) index provided in the CAT12 reports. The IQR index is calculated based on measures of noise, bias and image resolution, so we checked it to further ensure image quality. In this study, all images were above the satisfactory level of IQR (IQR index > 70%), which is consistent with the description of quality ratings in the CAT12 guidelines.

#### 2.3.4 Emotion Understanding Network

**FIGURE 2:**
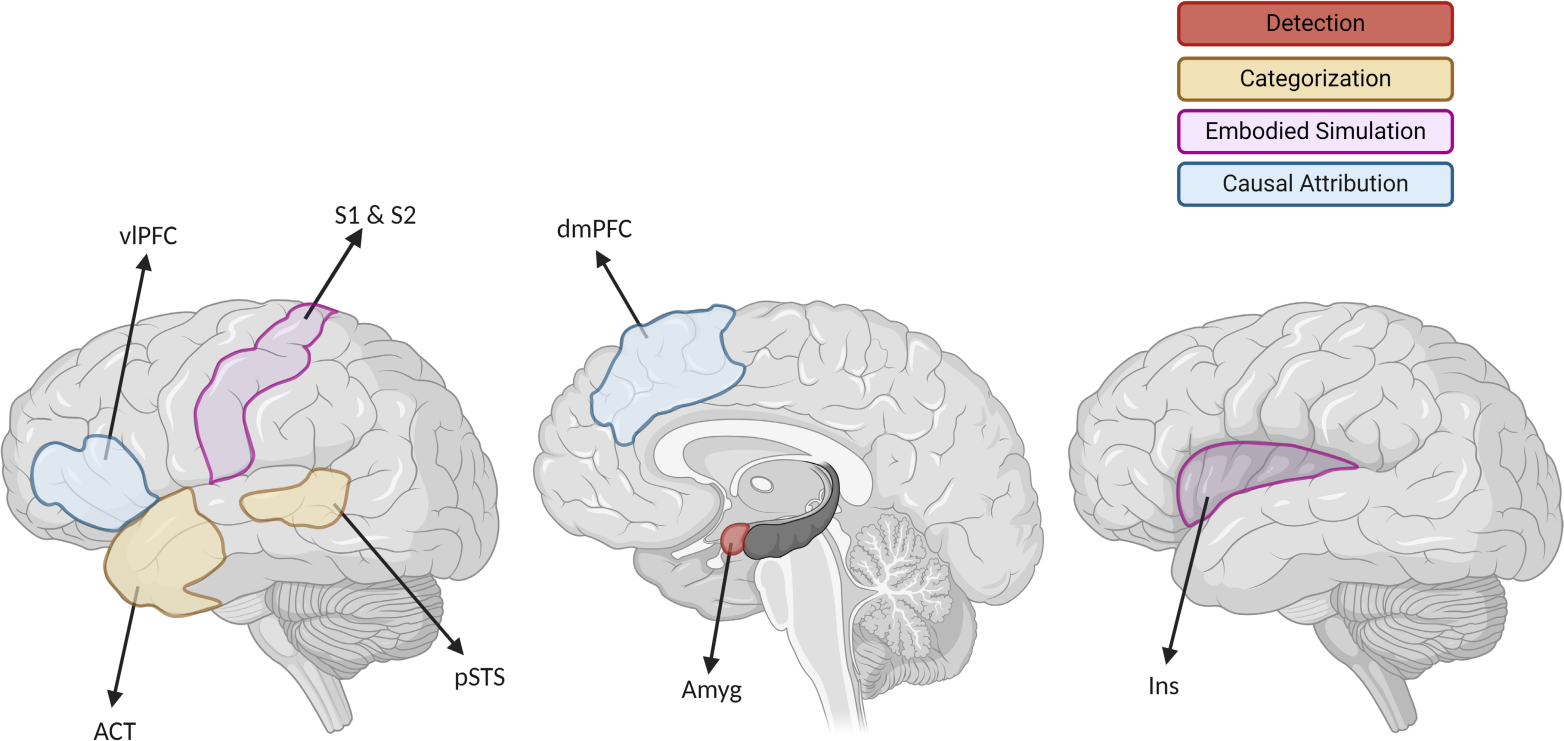
Brain regions associated with the different functional aspects of emotion understanding (adapted from Spunt and Adolphs 2019). Image created with BioRender.com (https://www.biorender.com, agreement number: QC26XF9BAG).

ROIs representing the emotion understanding network were selected based on the Spunt and Adolphs model (Spunt and Adolphs 2019) and derived from the Brainnetome Atlas (https://atlas.brainnetome.org/) (Fan et al. 2016). The ROIs included were the anterior temporal cortex (ATC), the posterior part of the superior temporal sulcus (pSTS), the primary and secondary somatosensory cortex (S1/S2), the insula (Ins), the amygdala (Amyg), the dorsomedial prefrontal cortex (dmPFC) and the ventrolateral prefrontal cortex (vlPFC). A total of 14 ROIs were considered, comprising both left‐ and right‐sided regions. See Figure 2 for a graphical representation.

### 2.4 Statistical Analyses

#### 2.4.1 Behavioural Data

PD patients were classified as impaired (PD‐IMP) or unimpaired (PD‐UN) based on their performance on the FACE test according to Italian normative data (Terruzzi et al. 2023). Specifically, regression‐based norms and equivalent scores (ES) derived according to the standardization procedure proposed by Capitani and Laiacona (Capitani and Laiacona 1997, 2017) were used to classify PD participants. Individuals with an adjusted FACE score <24.612 (Terruzzi et al. 2023) were included in the PD‐IMP group, while the others were in the PD‐UN group. This represents the standard method in Italy to evaluate the presence of a cognitive deficit in the context of a neuropsychological assessment. Group differences between PD‐IMP and PD‐UN patients in demographic, clinical, cognitive, and social characteristics were assessed using independent samples *t* test or Mann–Whitney *U* test based on data distribution. To account for possible differences in demographic variables between the two groups, the adjusted scores derived from the Italian normative data were used. The significance level was set at *p* < 0.05. All statistical analyses were performed with Jamovi 2.0.0 (Jamovi 2021; R Core Team 2021).

#### 2.4.2 MRI Data

All twenty‐four PD patients were included in the structural analyses, while two participants were excluded from the functional analyses due to excessive head movement during scanning (head movement >3 mm along any axis) or excessive mean framewise displacement [mean (FD); identified as an outlier with values greater than three scaled median absolute deviations from the median of the initial sample]. The results of the independent samples *t* test indicated that there were no significant differences between the PD‐UN and PD‐IMP subgroups in either mean head movement (*U* = 52, *p* = 1.00) or mean FD (*U* = 42, *p* = 0.49).

To investigate the association between FACE test performance and functional and structural changes within the emotion understanding network (Spunt and Adolphs 2019), ROI‐based analyses were conducted. At the functional level, *ROI‐to‐ROI connectivity* (RRC) matrices were estimated to characterize the patterns of FC associated with FACE test performance within the 14 ROIs of the emotion understanding network (Spunt and Adolphs 2019) (see ‘Regions of Interest’ section). At the structural level, GM intensities were extracted from these 14 ROIs in subject‐specific space during the pre‐processing steps.

To investigate the potential involvement of other brain regions outside our network of interest, whole‐brain exploratory analyses were also performed to complement the ROI results. Specifically, *seed‐based connectivity* (SBC) maps were generated to characterize FC patterns at a whole‐brain level, using the left and right amygdala separately as predefined seeds. This choice was based on previous evidence (Diederich et al. 2016) showing a relevant role of the amygdala in PD deficits, including dysfunction in emotion understanding. Instead, to examine the relationship between GM volume and the FACE test score, voxel‐wise structural analyses were performed.

Two different approaches were used for both ROI‐based and whole‐brain analyses. First, multiple regression analyses were used to investigate the relationship between both structural and functional changes in the PD sample and FACE performance. Nuisance variables included the MoCA adjusted score to control for patients' cognitive status and the Total Intracranial Volume (TIV) values (only in the structural analyses) to remove potential effects of brain size (Gaser et al. 2022). Furthermore, to assess potential possible differences between PD‐IMP and PD‐UN patients in terms of structural and functional characteristics, mean FC measures and GM values were contrasted between the two groups (two‐sample *T* test), controlling for MoCA adjusted score, sex and TIV (the latter only for the structural analyses).

The FC results were thresholded using a combination of a cluster‐forming *p* < 0.001 voxel‐level threshold, and a familywise corrected p‐FDR < 0.05 cluster‐size threshold. For GM results, the statistical significance level was set at *p* < 0.05 FDR‐corrected for ROI‐based analyses, while for whole‐brain analyses, the significance level was set at *p* < 0.001 (uncorrected) voxel‐wise and *p* < 0.05 FDR cluster‐level.

## 3 Results

### 3.1 Behavioural Data

The results of the statistical analyses revealed significant differences between the PD‐UN and PD‐IMP groups on the Unified Parkinson's Disease Rating Scale (UPDRS)–Part III (*t*(22) = −2.17; *p* = 0.04) and MoCA (*t*(22) = −2.74; *p* = 0.01). In particular, the PD‐IMP group exhibited more severe motor symptoms and poorer cognitive function than the PD‐UN group. Furthermore, the two groups were not balanced in terms of sex distribution (Fisher's exact test, *p* = 0.03). Table 1 presents the demographic, clinical, cognitive, and social characteristics of the two groups (PD‐UN vs. PD‐IMP). The FACE test score significantly correlated with MOCA (*r*_s_ = 0.45, *p* = 0.03), Hohen and Yahr scale (*r*_s_ = −0.66, *p* < 0.001) and with UPDRS part III (*r*_s_ = −0.44, *p* = 0.03).

**TABLE 1:** Between‐group comparisons for demographic, clinical, cognitive and social measures.

Variables	PD‐UN (*n* = 16)	PD‐IMP (*n* = 8)	Effect size	Statistics
Sex *(Female/Male)*	10/6	1/7	*ϕ* = 0.47	Fisher's exact test, ***p* = 0.03**
Age in years	65.2 ± 7.6	70.9 ± 3.4	*d* = −0.87	*t*(22) = −2.00; *p* = 0.06
Education in years	10.8 ± 3.4	10.9 ± 4.6	*d* = −0.03	*t*(22) = −0.08; *p* = 0.94
UPDRS—Part I	7.9 ± 4.3	8.0 ± 3.5	*d* = −0.02	*t*(22) = −0.04; *p* = 0.97
UPDRS—Part II	6.5 ± 2.9	9.0 ± 6.1	*d* = −0.60	*t*(22) = −1.38; *p* = 0.18
UPDRS—Part III	16.3 ± 7.1	24.4 ± 11.1	*d* = −0.94	*t*(22) = −2.17; ***p* = 0.04**
UPDRS—Part IV	1.8 ± 3.2	1.0 ± 1.4	*rₓ* = 0.04	*U* = 62; *p* = 0.88
Disease duration in months	65.2 ± 38.4	55.5 ± 26.4	*d* = 0.28	*t*(22) = 0.65; *p* = 0.52
Hoehn and Yahr Stage scale	1.6 ± 0.7	2.1 ± 0.6	*d* = −0.81	*t*(22) = −1.87; *p* = 0.08
LEDD	433.1 ± 289.2	570.3 ± 237.0	*rₓ* = 0.32	*U* = 31; *p* = 0.27
MoCA^a^	23.7 ± 3.4	19.7 ± 3.6	*d* = 1.19	*t*(22) = 2.74; ***p* = 0.01**
FACE test^a^	31.0 ± 2.1	21.0 ± 2.2	*d* = 4.60	*t*(22) = 10.63; ***p* < 0.001**

*Note:* Data represented as mean ± standard deviation. Effect sizes reported as Cohen's *d* for parametric tests, Rank Biserial Correlation (*rₓ*) for non‐parametric tests and Phi coefficient (*ϕ*) for Chi‐squared statistic. UPDRS: Unified Parkinson's Disease Rating Scale, LEDD: Levodopa equivalent daily dose; MoCA: Montreal Cognitive Assessment, FACE: FAcial Complex Expressions; PD‐UN: classified as unimpaired based on their performance on the FACE test according to Italian normative data; PD‐IMP: PD patients classified as impaired based on their performance on the FACE test according to Italian normative data.

^a^ Adjusted scores based on Italian normative data.

### 3.2 Emotion Processing According to Spunt and Adolphs Model—ROI‐Based Analyses

**FIGURE 3:**
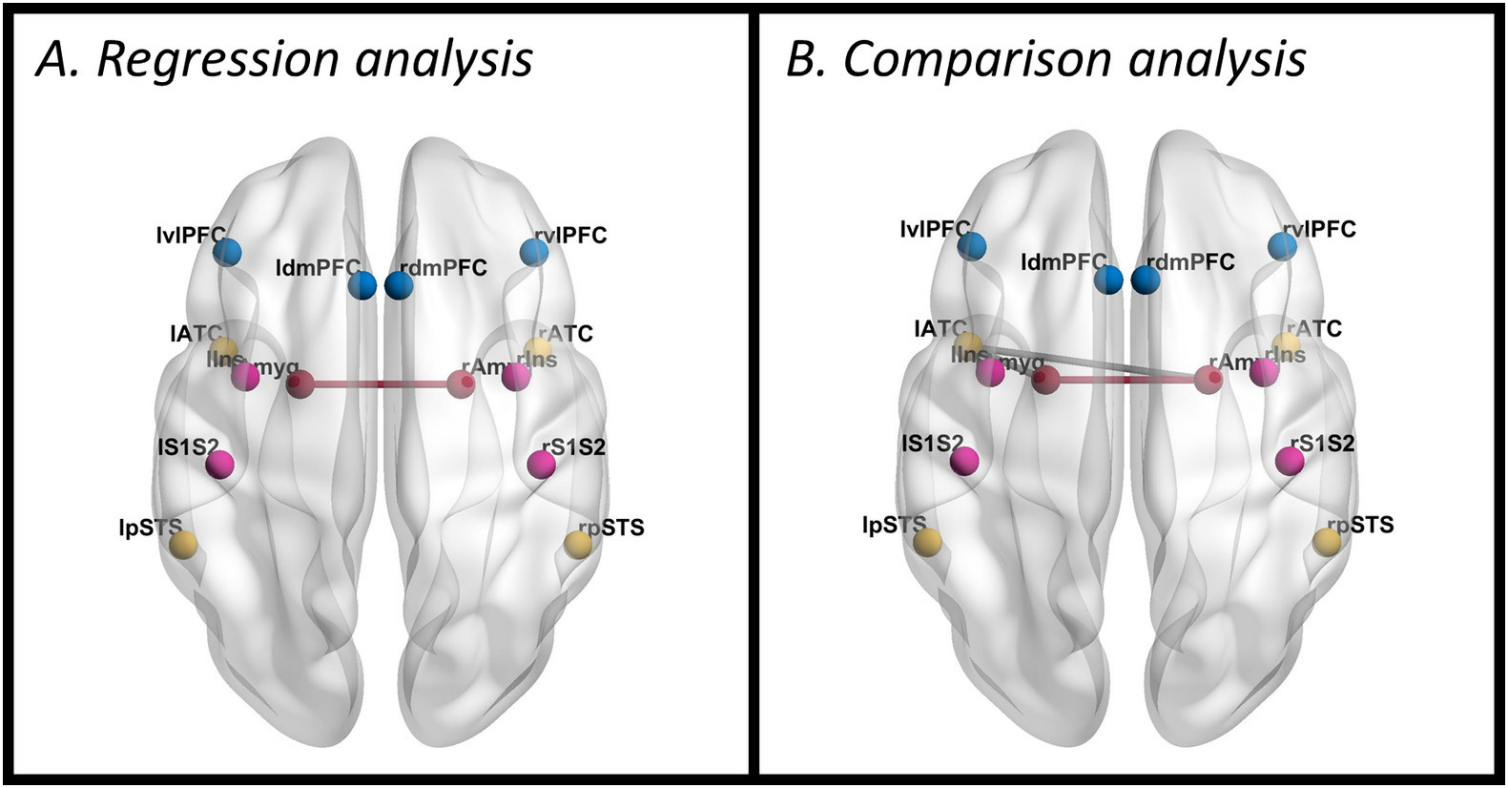
ROI‐based FC results—(A) Significant positive association between ROI‐to‐ROI FC metrics and FACE test score in PD patients. (B) Difference in average connectivity between PD‐UN and PD‐IMP groups. Images created with BrainNet Viewer (http://www.nitrc.org/projects/bnv/) (Xia, Wang, and He 2013).

At the functional level, the *RRC* matrix among 14 ROIs (91 connections sorted using hierarchical clustering) across the whole PD sample revealed a positive association between FACE test performance and BOLD co‐activation of the right and left amygdala (*t*(19) = 4.48, p‐FDR = 0.003) (Figure 3A).

Besides, the results of the comparative RRC analysis between PD‐UN and PD‐IMP showed that PD patients impaired on the FACE test had lower FC than unimpaired PD patients between left and right amygdala (*t*(18) = −5.60, p‐FDR = 0.0003), left amygdala and left anterior temporal cortex (*t*(18) = −4.93, p‐FDR = 0.0007) and right amygdala and left anterior temporal cortex (*t*(18) = −4.27, p‐FDR = 0.0030) (Figure 3B). No significant regions were identified with higher functional connectivity (FC).

**FIGURE 4:**
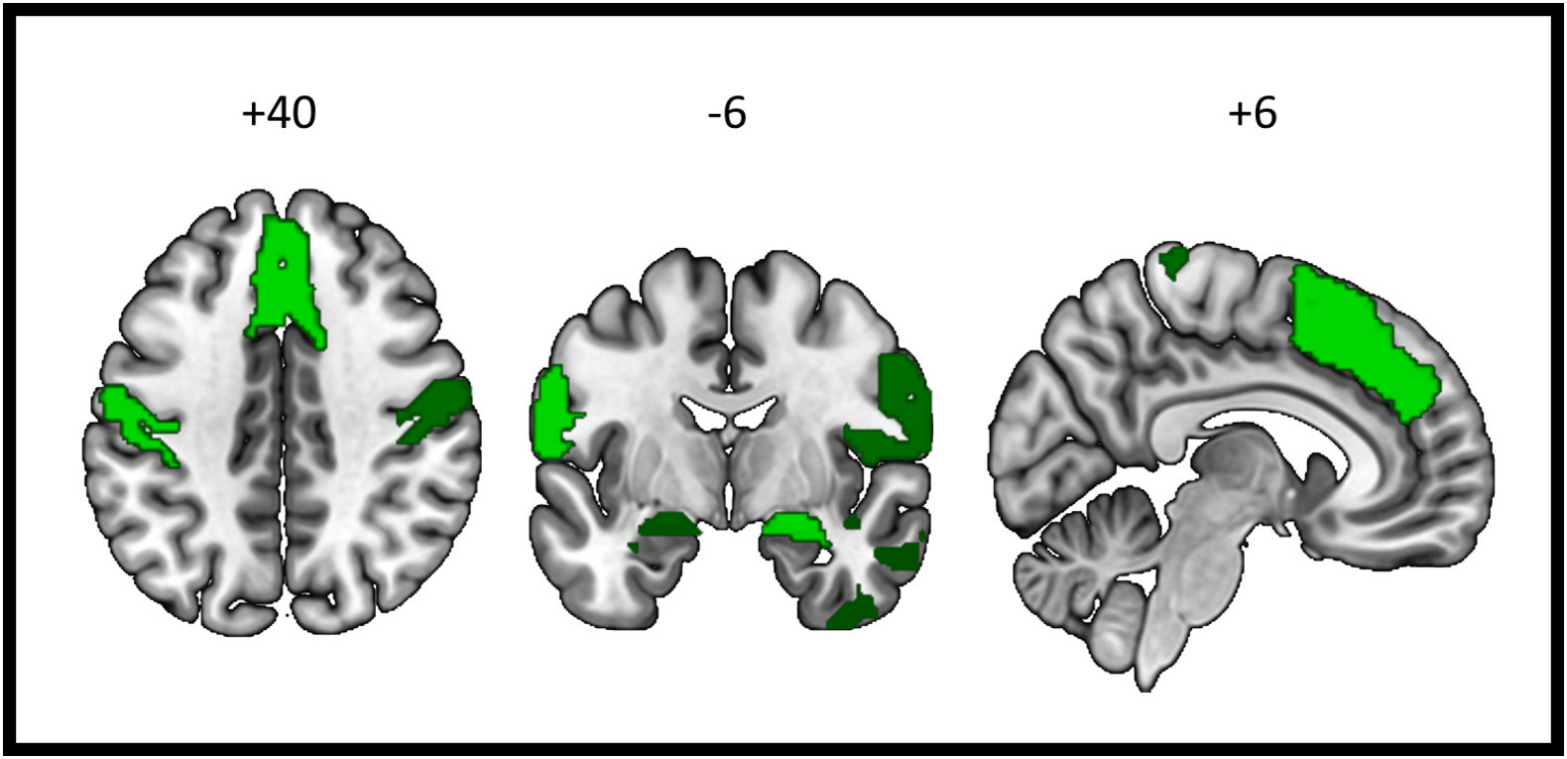
ROI‐based VBM results—Significant positive association between GM volume and FACE test score in PD patients. Colour scale indicates *t* statistic: light green, *t* value >3; dark green, *t* value >2. Significance: *p* < 0.05 FDR corrected. Images displayed in neurological convention.

At the structural level, ROI‐based multiple regression analysis across the entire PD sample on 14 ROIs involved in emotion understanding showed a significant effect of FACE test score in bilateral dorsomedial prefrontal cortex, bilateral primary and secondary somatosensory cortex, bilateral amygdala, and right anterior temporal cortex (see Figure 4 and Table 2). A significant difference also emerged between the two groups (PD‐IMP vs PD‐UN) only in the right amygdala (*p* value uncorrected = 0.0057, *t* value = 2.82), where the PD‐UN group had higher GM volume than the PD‐IMP.

**TABLE 2:** ROI‐based VBM results—Significant positive association with GM volume in PD patients.

Regions of interest	Uncorrected	FDR‐corrected	*t* value
	*p*	*p*	
*L dorsomedial prefrontal cortex*	0.0025	0.0146	3.16
*L ventrolateral prefrontal cortex*	0.0326	—	1.95
*L primary & secondary somatosensory cortex*	0.0042	0.0146	2.93
*L amygdala*	0.0189	0.0440	2.23
*L anterior temporal cortex*	0.0336	—	1.94
*R dorsomedial prefrontal cortex*	0.0034	0.0146	3.01
*R primary and secondary somatosensory cortex*	0.0138	0.0386	2.38
*R amygdala*	0.0017	0.0146	3.33
*R anterior temporal cortex*	0.0242	0.0484	2.10

*Note:* Both FDR‐corrected and uncorrected results are displayed.

### 3.3 Exploratory Analyses at Whole‐Brain Level

**FIGURE 5:**
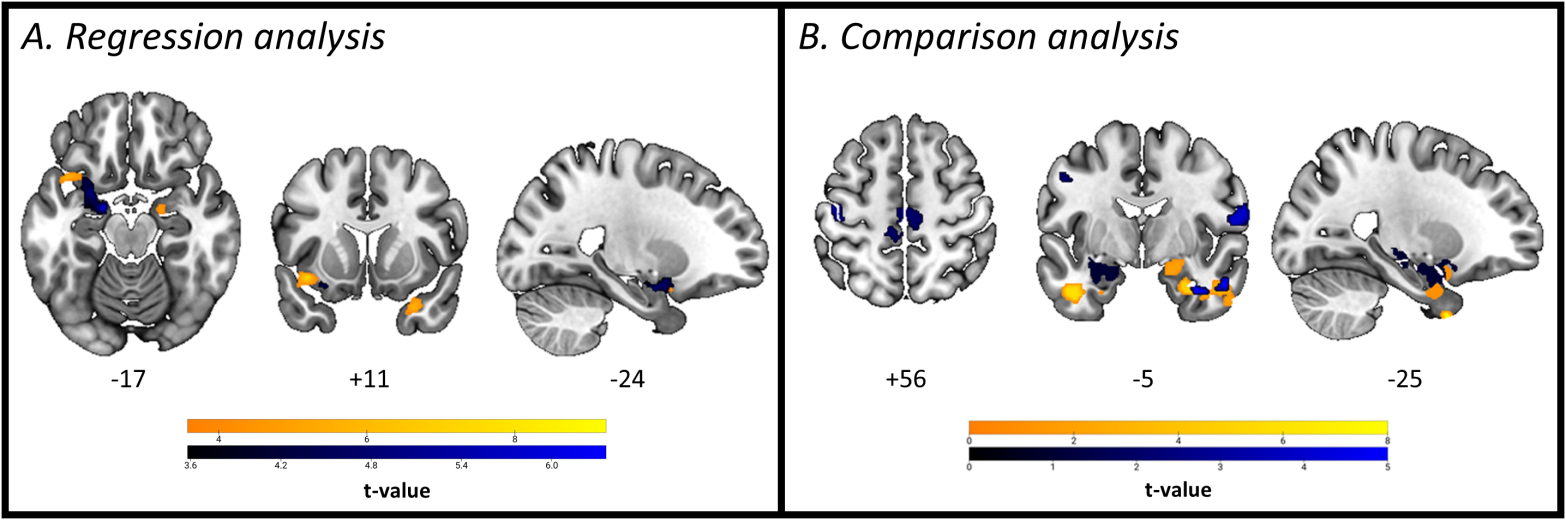
Whole‐brain FC results—(A) SBC maps associated with FACE test score in PD patients. (B) SBC maps comparing PD‐UN and PD‐IMP*.* Colours indicate connections from left (yellow) and right (blue) amygdala to the whole brain. Images displayed in neurological convention.

At the functional level, further exploratory SBC regression analyses using the left and right amygdala separately as seeds revealed significant positive correlations between the FACE test performance and FC between the left amygdala and both the right temporal pole/right amygdala (*x* = 30, *y* = 10, *z* = −30; p‐FDR = 0.0258) and the left insular cortex (*x* = −40, *y* = 14, *z* = −12; p‐FDR = 0.0258), as well as between the right–left amygdala (*x* = −18; *y* = −06; *z* = −18; p‐FDR = 0.0151) across the entire PD sample (Figure 5A). On the other hand, results of comparative SBC analyses between PD‐UN and PD‐IMP showed a difference in mean FC between bilateral amygdala and bilateral temporal cluster (including inferior and middle temporal gyrus, temporal pole), as well as between right amygdala and bilateral pre/post‐central gyrus, with PD‐IMP group showing reduced FC (Figure 5B and Table 3). No increased FC was found in the PD‐IMP compared to the PD‐UN subgroup.

**TABLE 3:** Whole‐brain FC results—SBC analysis of left/right amygdala comparing PD‐UN and PD‐IMP.

Probabilistic anatomical label	Cluster (*x*,*y*,*z*)	Uncorrected	FDR‐corrected	*t* value
		*p*	*p*	
***Seed—Left Amygdala***				
*L Temporal Pole*	−40,+14,−12	0.000002	0.000077	9.81
*R Hippocampus/Right Amygdala*	+24,−26,−12	0.000088	0.001409	7.61
*R Inferior Temporal Gyrus, posterior division*	+50,−8,−28	0.000278	0.002433	6.22
*L Middle Temporal Gyrus, posterior division*	−64,−28,−8	0.000359	0.002433	5.89
*R Temporal Pole*	+28,−6,−30	0.000380	0.002433	5.78
*L Inferior Temporal Gyrus, anterior division*	−46,−8,−30	0.001479	0.007888	5.77
*L Supramarginal Gyrus, posterior division*	−42,−48,+10	0.004734	0.021641	5.54
*L Temporal Pole*	−40,+2,−44	0.005647	0.022589	5.12
***Seed—Right Amygdala***				
*L Amygdala*	−18,−6,−20	0.000001	0.000036	9.04
*L/R Precentral Gyrus*	+6,−22,+ 54	0.000004	0.000104	6.78
*L Precentral and Postcentral Gyrus*	−40,−18,+42	0.000007	0.000135	6.37
*R Postcentral Gyrus*	+66,−10,+14	0.001917	0.027314	5.80
*R Inferior Temporal Gyrus, posterior division*	+52,−8,−28	0.002688	0.030648	5.04

*Note:* Coordinates for each cluster that exceeds the thresholds specified in the text, showing a higher FC in the PD‐UN versus PD‐IMP group.

At the structural level, VBM multiple regression analysis performed at the whole‐brain level showed a significant cluster spanning the dorsomedial and the ventromedial prefrontal cortex (peak MNI coordinates: *x* = 0; *y* = 45; *z* = 15; *k* = 4109; *p* < 0.05 FDR corrected at cluster level; *t* value = 6.72) across the entire PD sample. When comparing PD‐IMP and PD‐UN, no results survived FDR correction. Using an uncorrected threshold at voxel‐level (*p* < 0.001), the differences between impaired and unimpaired PD patients were located in the dorsomedial prefrontal cortex (paracingulate and cingulate gyrus, x = 0, *y* = 45, *z* = 14; p‐unc < 0.001, *t* value = 5.83; middle frontal gyrus, *x* = −36, *y* = 11, *z* = 36, p‐unc = 0.001, *t* value = 3.65), left lateral occipital cortex (*x* = −32, *y* = −86, *z* = 44; p‐unc < 0.001, *t* value = 4.38) and right temporal pole/amygdala (*x* = 27, *y* = 5, *z* = −29; p‐unc = 0.001, *t* value = 3.70).

## 4 Discussion

A substantial body of evidence suggests that individuals diagnosed with PD exhibit a range of emotional processing deficits, from emotional experience to emotion production and recognition (Argaud et al. 2018; Péron et al. 2012). Nevertheless, despite mounting clinical evidence of socio‐cognitive dysfunction in this population, the neural correlates underlying altered emotional processing in PD remain largely undefined. Overall, emotional processing deficits may arise from the dysfunction of the amygdala in PD (Diederich et al. 2016; Tessitore et al. 2002; Yoshimura et al. 2005) and the impairment of the cortico‐striatal‐thalamic‐cortical loop, which is modulated by the mesolimbic dopamine system and is affected by PD pathology (Péron et al. 2012). However, the few neuroimaging studies currently available on PD patients have focused on the recognition of basic emotions (Baggio et al. 2012; Ibarretxe‐Bilbao et al. 2009; Tessitore et al. 2002; Wabnegger et al. 2015), with no evidence for complex mental states. The present study aimed to address this gap by investigating the neural correlates of facial complex mental state recognition from faces in non‐demented PD patients using a multi‐level neuroimaging approach. Specifically, the association between FACE test performance and both resting‐state FC and GM volumes in PD patients was tested within a defined network critical for emotion understanding (Spunt and Adolphs 2019) and at the whole‐brain level. Furthermore, we compared FC and GM values in PD patients with and without clinical deficits on the FACE test according to Italian normative data (Terruzzi et al. 2023).

The results of this study indicate that the bilateral amygdala plays a critical role in the recognition of complex mental states from facial expressions in PD patients at both the structural and functional levels. This is consistent with previous theoretical models proposing an early amygdala impairment in PD associated with altered processing of social signals (Argaud et al. 2018; Diederich et al. 2016; Wang et al. 2023). In particular, Diederich et al. (2016) emphasized the compromised recognition of emotional facial expressions as a prominent symptom of amygdala network dysfunction since the early stages of PD, along with impaired theory of mind and hypomimia.

While previous structural (Baggio et al. 2012; Ibarretxe‐Bilbao et al. 2009) and functional (Tessitore et al. 2002) studies in PD have documented the impairment of the amygdala in basic emotion recognition, its involvement in complex emotion recognition remained unexplored. Consequently, our study offers novel insights by emphasizing the significance of this hub in the overall detection of emotion‐relevant sensory stimuli.

Furthermore, the comparison of PD individuals with and without deficits in mental state recognition also revealed significant alterations in the patterns of FC between the bilateral amygdalae and the left anterior temporal cortex, which extended to the right anterior temporal cortex in the structural analyses. This result is consistent with the recent acknowledgement in the literature of the bilateral anterior temporal lobe as a crucial hub for social‐semantic knowledge (Olson, Plotzker, and Ezzyat 2007; Rouse et al. 2024; Thye, Hoffman, and Mirman 2024), thanks to its structural connections with limbic structures (Olson et al. 2013).

The findings from functional whole‐brain analyses confirmed the involvement of regions belonging to the emotion understanding network (Spunt and Adolphs 2019) (the amygdala, the temporal cortex, and the insula) in the recognition of complex mental states. Interestingly, the insula did not emerge in the ROI‐based analysis among the areas involved in FACE performance. However, it is worthy of attention that at the ROI level we did not distinguish between different sub‐portions (i.e., anterior or posterior), while in the whole‐brain analysis, we observed a specific involvement of the anterior portion. This is consistent with the well‐known evidence supporting a prominent role of the anterior insula in emotional awareness, compared to the posterior part, dedicated to primary interoceptive representation (Craig 2009; Kurth et al. 2010). Furthermore, the aforementioned analyses demonstrated the involvement of additional temporal areas not included in the Spunt and Adolphs model (Spunt and Adolphs 2019), namely, the inferior/middle temporal gyri bilaterally. The extensive engagement of temporal areas in the processing of both mental states in general (Abu‐Akel and Shamay‐Tsoory 2011), and specific features relevant to social cognitive processing, such as faces (Sellal 2022), is required in FACE test performance and can justify these findings.

The structural imaging results confirmed the pivotal role of the amygdala in emotion processing and demonstrated a positive association between FACE performance and GM volumes in other brain areas included in the Spunt and Adolphs (2019) model, namely, the dorsomedial prefrontal cortex and the primary/secondary somatosensory cortex bilaterally. Although neuroimaging studies in PD patients are still relatively limited in scope, preliminary evidence suggests that the medial prefrontal activation (Moonen et al. 2017) and the somatosensory recruitment (Wabnegger et al. 2015) may be involved in compensatory top‐down cognitive control mechanisms, restoring striatal dysfunction related to the disease's pathology and modulating emotion regulation in PD patients. Collectively, these compensatory mechanisms could explain why we observed a positive association with the FACE test without GM reduction at the structural level in our PD sample, as previously suggested. However, the absence of a control group prevents us from ruling out the possibility of greater or lesser activation in these brain areas in relation to complex mental state recognition. Further research is required to more fully elucidate this aspect.

Despite the encouraging outcomes, it is essential to acknowledge the limitations of our study, which may influence our findings and suggest areas for future research. The principal methodological constraints of the present study are the relatively modest sample size and the absence of a healthy control group. Nevertheless, the inclusion of PD individuals without socio‐cognitive dysfunctions as a comparison group, and the evidence of significant differences between this group and PD‐IMP patients at the brain level, allowed us to partially overcome this limitation. Moreover, the test has been already clinically validated in PD patients, showing high diagnostic accuracy in discriminating PD individuals with and without socio‐cognitive dysfunctions (Terruzzi et al. 2023). In addition, some patients had low scores at the MoCA test. However, these data should be interpreted taking into account the lower Italian cut‐off for this screening test (Conti et al. 2015), compared to that adopted in the international scenario.

## 5 Conclusion

In conclusion, the results of the present study indicate that complex mental state recognition from facial expressions in PD patients is associated with structural and functional changes in brain areas involved in emotion understanding (Spunt and Adolphs 2019), at both cortical and subcortical levels. In particular, the functional findings highlight the relevant role of the bilateral amygdala, in accordance with previous theoretical models (Diederich et al. 2016). In addition, anatomical modifications have been identified in other brain areas involved in emotional cue detection (i.e., the amygdala), emotion categorization and maintenance of semantic knowledge about the social world (the anterior temporal lobes), embodied simulation (the primary and secondary somatosensory cortex) and causal attribution (the dorsomedial prefrontal cortex). Furthermore, the results of the whole‐brain analyses extend the neural network associated with the recognition of complex mental states in PD patients to the medial prefrontal regions, which are known to be highly involved in the interaction between cognitive and emotional processes in the brain (Ray and Zald 2012). Collectively, these findings contribute to providing valuable insights into the neural underpinnings of complex mental state recognition in PD. Moreover, the characterization of early socio‐cognitive dysfunctions in a subsample of individuals may facilitate the definition of new cognitive phenotypes (Czernecki et al. 2021; Dodich et al. 2022; Pagonabarraga et al. 2015), and potentially the development of new non‐pharmacological interventions tailored to the specific needs of the individual.

## Author Contributions

**Funghi Giulia:** conceptualization, data curation, formal analysis, investigation, methodology, visualization, writing – original draft, writing – review and editing. **Rabini Giuseppe:** conceptualization, data curation, formal analysis, investigation, methodology, software, supervision, validation, writing – review and editing. **Meli Claudia:** data curation, investigation, writing – review and editing. **Speranza Chiara:** data curation, formal analysis, investigation, software, visualization, writing – original draft, writing – review and editing. **Pierotti Enrica:** data curation, investigation, writing – review and editing. **Saviola Francesca:** data curation, investigation, writing – review and editing. **Tambalo Stefano:** data curation, investigation, validation, writing – review and editing. **Zappini Francesca:** data curation, investigation, writing – review and editing. **Fumagalli Giorgio:** data curation, investigation, writing – review and editing. **Turella Luca:** conceptualization, data curation, formal analysis, funding acquisition, investigation, methodology, project administration, resources, supervision, validation, visualization, writing – original draft, writing – review and editing. **Jovicich Jorge:** data curation, investigation, project administration, resources, writing – review and editing. **Papagno Costanza:** data curation, investigation, project administration, resources, supervision, validation, writing – review and editing. **Dodich Alessandra:** conceptualization, data curation, formal analysis, investigation, methodology, project administration, resources, supervision, validation, visualization, writing – original draft, writing – review and editing.

## Ethics Statement

All procedures were performed in compliance with relevant laws and institutional guidelines and approved by the Ethical Committee of the University of Trento (N. prot. 2019‐033). The study was conducted following the World Medical Association Declaration of Helsinki: Ethical principles for medical research involving human subjects.

Privacy rights of human subjects have been observed and written informed consent for participation in the study was obtained from all participants and included the possibility of publication of the data obtained in the study.

All authors confirm that they have read the journal's position on issues involved in ethical publication and affirm that this work is consistent with those guidelines.

## Conflicts of Interest

The authors declare no conflicts of interest.

### Peer Review

The peer review history for this article is available at https://www.webofscience.com/api/gateway/wos/peer‐review/10.1111/ejn.70014.

## Data Availability

Datasets generated and analysed in this study are available from the corresponding author on request.

## References

[ejn70014-bib-0001] Abu‐Akel, A. , and S. Shamay‐Tsoory . 2011. “Neuroanatomical and Neurochemical Bases of Theory of Mind.” Neuropsychologia 49, no. 11: 2971–2984. 10.1016/J.NEUROPSYCHOLOGIA.2011.07.012. 21803062

[ejn70014-bib-0002] Argaud, S. , M. Vérin , P. Sauleau , and D. Grandjean . 2018. “Facial Emotion Recognition in Parkinson's Disease: A Review and New Hypotheses.” Movement Disorders 33, no. 4: 554–567. 10.1002/MDS.27305. 29473661 PMC5900878

[ejn70014-bib-0003] Baggio, H. C. , B. Segura , N. Ibarretxe‐Bilbao , et al. 2012. “Structural Correlates of Facial Emotion Recognition Deficits in Parkinson's Disease Patients.” Neuropsychologia 50, no. 8: 2121–2128. 10.1016/J.NEUROPSYCHOLOGIA.2012.05.020. 22640663

[ejn70014-bib-0004] Bell, P. T. , M. Gilat , J. M. Shine , K. L. McMahon , S. J. G. Lewis , and D. A. Copland . 2019. “Neural Correlates of Emotional Valence Processing in Parkinson's Disease: Dysfunction in the Subcortex.” Brain Imaging and Behavior 13, no. 1: 189–199. 10.1007/s11682-017-9754-3. 28812218

[ejn70014-bib-0005] Benzagmout, M. , S. Boujraf , B. Alami , et al. 2019. “Emotion Processing in Parkinson's Disease: A Blood Oxygenation Level‐Dependent Functional Magnetic Resonance Imaging Study.” Neural Regeneration Research 14, no. 4: 666–672. 10.4103/1673-5374.247470. 30632507 PMC6352597

[ejn70014-bib-0006] Bodden, M. E. , R. Dodel , and E. Kalbe . 2010. “Theory of Mind in Parkinson's Disease and Related Basal Ganglia Disorders: A Systematic Review.” Movement Disorders 25, no. 1: 13–27. 10.1002/mds.22818. 19908307

[ejn70014-bib-0007] Bora, E. , M. Walterfang , and D. Velakoulis . 2015. “Theory of Mind in Parkinson's Disease: A Meta‐Analysis.” Behavioural Brain Research 292: 515–520. 10.1016/J.BBR.2015.07.012. 26166188

[ejn70014-bib-0008] Capitani, E. , and M. Laiacona . 1997. “Normative Data and Neuropsychological Assessment. Common Problems in Clinical Practice and Research.” Neuropsychological Rehabilitation 7, no. 4: 295–310. 10.1080/713755543.

[ejn70014-bib-0009] Capitani, E. , and M. Laiacona . 2017. “Outer and Inner Tolerance Limits: Their Usefulness for the Construction of Norms and the Standardization of Neuropsychological Tests.” Clinical Neuropsychologist 31, no. 6–7: 1219–1230. 10.1080/13854046.2017.1334830. 28598726

[ejn70014-bib-0010] Conti, S. , S. Bonazzi , M. Laiacona , M. Masina , and M. V. Coralli . 2015. “Montreal Cognitive Assessment (MoCA)‐Italian Version: Regression Based Norms and Equivalent Scores.” Neurological Sciences 36, no. 2: 209–214. 10.1007/s10072-014-1921-3. 25139107

[ejn70014-bib-0011] Coundouris, S. P. , A. G. Adams , and J. D. Henry . 2020. “Empathy and Theory of Mind in Parkinson's Disease: A Meta‐Analysis.” Neuroscience and Biobehavioral Reviews 109: 92–102. 10.1016/j.neubiorev.2019.12.030. 31899300

[ejn70014-bib-0012] Craig, A. D. B. 2009. “How Do You Feel‐‐Now? The Anterior Insula and Human Awareness.” Nature Reviews. Neuroscience 10, no. 1: 59–70. 10.1038/nrn2555. 19096369

[ejn70014-bib-0013] Czernecki, V. , E. Benchetrit , M. Houot , et al. 2021. “Social Cognitive Impairment in Early Parkinson's Disease: A Novel “Mild Impairment”?” Parkinsonism & Related Disorders 85: 117–121. 10.1016/j.parkreldis.2021.02.023. 33812772

[ejn70014-bib-0014] Diederich, N. J. , J. G. Goldman , G. T. Stebbins , and C. G. Goetz . 2016. “Failing as Doorman and Disc Jockey at the Same Time: Amygdalar Dysfunction in Parkinson's Disease.” Movement Disorders 31, no. 1: 11–22. 10.1002/MDS.26460. 26650182

[ejn70014-bib-0015] Dodich, A. , G. Funghi , C. Meli , et al. 2022. “Deficits in Emotion Recognition and Theory of Mind in Parkinson's Disease Patients With and Without Cognitive Impairments.” Frontiers in Psychology 13: 866809. 10.3389/fpsyg.2022.866809. 35645902 PMC9138611

[ejn70014-bib-0016] Emre, M. , D. Aarsland , R. Brown , et al. 2007. “Clinical Diagnostic Criteria for Dementia Associated With Parkinson's Disease.” Movement Disorders 22, no. 12: 1689–1707. 10.1002/mds.21507. 17542011

[ejn70014-bib-0017] Enrici, I. , M. Adenzato , R. B. Ardito , et al. 2015. “Emotion Processing in Parkinson's Disease: A Three‐Level Study on Recognition, Representation, and Regulation.” PLoS ONE 10, no. 6: e0131470. 10.1371/JOURNAL.PONE.0131470. 26110271 PMC4482447

[ejn70014-bib-0018] Fan, L. , H. Li , J. Zhuo , et al. 2016. “The Human Brainnetome Atlas: A New Brain Atlas Based on Connectional Architecture.” Cerebral Cortex 26: 3508–3526. 10.1093/cercor/bhw157. 27230218 PMC4961028

[ejn70014-bib-0019] Frith, C. D. 2007. “The Social Brain?” Philosophical Transactions of the Royal Society, B: Biological Sciences 362, no. 1480: 671–678. 10.1098/rstb.2006.2003. PMC1919402 17255010

[ejn70014-bib-0020] Gaser, C. , R. Dahnke , P. M. Thompson , F. Kurth , C. Gaser , and D. Ph . 2022. “CAT – A Computational Anatomy Toolbox for the Analysis of Structural MRI.” In *Biorxiv*. 10.1093/gigascience/giae049 PMC11299546 39102518

[ejn70014-bib-0021] Gray, H. M. , and L. Tickle‐Degnen . 2010. “A Meta‐Analysis of Performance on Emotion Recognition Tasks in Parkinson's Disease.” Movement Disorders 24, no. 2: 176–191. 10.1037/A0018104. 20230112

[ejn70014-bib-0022] Happé, F. , J. L. Cook , and G. Bird . 2017. “The Structure of Social Cognition: In (ter)dependence of Sociocognitive Processes.” Annual Review of Psychology 68: 243–267. 10.1146/annurev-psych-010416-044046. 27687121

[ejn70014-bib-0023] Hoehn, M. M. , and M. D. Yahr . 1967. “Parkinsonism: Onset, Progression and Mortality.” Neurology 17, no. 5: 427–442. 10.1212/WNL.17.5.427. 6067254

[ejn70014-bib-0024] Hughes, A. J. , S. E. Daniel , L. Kilford , and A. J. Lees . 1992. “Accuracy of Clinical Diagnosis of Idiopathic Parkinson's Disease: A Clinico‐Pathological Study of 100 Cases.” Journal of Neurology, Neurosurgery, and Psychiatry 55, no. 3: 181–184. 10.1136/JNNP.55.3.181. 1564476 PMC1014720

[ejn70014-bib-0025] Ibarretxe‐Bilbao, N. , C. Junque , E. Tolosa , et al. 2009. “Neuroanatomical Correlates of Impaired Decision‐Making and Facial Emotion Recognition in Early Parkinson's Disease.” European Journal of Neuroscience 30, no. 6: 1162–1171. 10.1111/J.1460-9568.2009.06892.X. 19735293

[ejn70014-bib-0026] Jamovi . 2021. ”The Jamovi Project. (Version 2.0) [Computer Software].” https://www.jamovi.org/.

[ejn70014-bib-0027] Kurth, F. , K. Zilles , P. T. Fox , A. R. Laird , and S. B. Eickhoff . 2010. “A Link Between the Systems: Functional Differentiation and Integration Within the Human Insula Revealed by Meta‐Analysis.” Brain Structure & Function 214, no. 5–6: 519–534. 10.1007/s00429-010-0255-z. 20512376 PMC4801482

[ejn70014-bib-0028] Lewis, S. J. G. , and L. Ricciardi . 2021. “Social Cognition in Parkinson's Disease.” Parkinsonism & Related Disorders 85: 122–123. 10.1016/j.parkreldis.2021.02.024. 33640252

[ejn70014-bib-0029] Moonen, A. J. H. , P. H. Weiss , M. Wiesing , et al. 2017. “An fMRI Study Into Emotional Processing in Parkinson's Disease: Does Increased Medial Prefrontal Activation Compensate for Striatal Dysfunction?” PLoS ONE 12, no. 5: e0177085. 10.1371/journal.pone.0177085. 28486506 PMC5423613

[ejn70014-bib-0030] Nieto‐Castanon, A. 2021. ”CONN Functional Connectivity Toolbox (RRID: SCR_009550), Version 21.” 10.56441/hilbertpress.2161.7292.

[ejn70014-bib-0031] Obeso, J. A. , C. Marin , C. Rodriguez‐Oroz , et al. 2008. “The Basal Ganglia in Parkinson's Disease: Current Concepts and Unexplained Observations.” Annals of Neurology 64, no. Suppl.2: S30–S46. 10.1002/ANA.21481. 19127584

[ejn70014-bib-0032] Olson, I. R. , D. McCoy , E. Klobusicky , and L. A. Ross . 2013. “Social Cognition and the Anterior Temporal Lobes: A Review and Theoretical Framework.” Social Cognitive and Affective Neuroscience 8, no. 2: 123–133. 10.1093/scan/nss119. 23051902 PMC3575728

[ejn70014-bib-0033] Olson, I. R. , A. Plotzker , and Y. Ezzyat . 2007. “The Enigmatic Temporal Pole: A Review of Findings on Social and Emotional Processing.” Brain 130, no. 7: 1718–1731. 10.1093/brain/awm052. 17392317

[ejn70014-bib-0034] Pagonabarraga, J. , J. Kulisevsky , A. P. Strafella , and P. Krack . 2015. “Apathy in Parkinson's Disease: Clinical Features, Neural Substrates, Diagnosis, and Treatment.” Lancet Neurology 14, no. 5: 518–531. 10.1016/S1474-4422(15)00019-8. 25895932

[ejn70014-bib-0035] Palmeri, R. , V. Lo Buono , F. Corallo , et al. 2017. “Nonmotor Symptoms in Parkinson Disease: A Descriptive Review on Social Cognition Ability.” Journal of Geriatric Psychiatry and Neurology 30, no. 2: 109–121. 10.1177/0891988716687872. 28073327

[ejn70014-bib-0036] Penny, W. D. , K. J. Friston , J. T. Ashburner , S. J. Kiebel , and T. E. Nichols . 2011. Statistical Parametric Mapping: The Analysis of Functional Brain Images. Amsterdam, Netherlands: Elsevier.

[ejn70014-bib-0037] Péron, J. , T. Dondaine , F. Le Jeune , D. Grandjean , and M. Vérin . 2012. “Emotional Processing in Parkinson's Disease: A Systematic Review.” Movement Disorders 27, no. 2: 186–199. 10.1002/mds.24025. 22162004

[ejn70014-bib-0038] R Core Team . 2021. “R: A Language and Environment for Statistical Computing. (Version 4.0) [Computer Software].” (R packages retrieved from MRAN snapshot 2021‐04‐01), https://cran.r‐project.org.

[ejn70014-bib-0039] Rabini, G. , G. Funghi , C. Meli , et al. 2023. “Functional Alterations in Resting‐State Networks for Theory of Mind in Parkinson's Disease.” European Journal of Neuroscience 59: 1213–1226. 10.1111/ejn.16145. 37670685

[ejn70014-bib-0040] Ray, R. D. , and D. H. Zald . 2012. “Anatomical Insights Into the Interaction of Emotion and Cognition in the Prefrontal Cortex.” Neuroscience and Biobehavioral Reviews 36: 479–501. 10.1016/j.neubiorev.2011.08.005. 21889953 PMC3244208

[ejn70014-bib-0041] Rouse, M. A. , R. J. Binney , K. Patterson , J. B. Rowe , and M. A. Lambon Ralph . 2024. “A Neuroanatomical and Cognitive Model of Impaired Social Behaviour in Frontotemporal Dementia.” Brain: A Journal of Neurology 147: 1953–1966. 10.1093/brain/awae040. 38334506 PMC11146431

[ejn70014-bib-0042] Schwartz, R. , D. Zulman , C. Gray , M. K. Goldstein , and R. Trivedi . 2020. ““It's a Disease of Families”: Neurologists' Insights on How to Improve Communication and Quality of Life for Families of Parkinson's Disease Patients.” Chronic Illness 16, no. 3: 201–211. 10.1177/1742395318799852. 30208725

[ejn70014-bib-0043] Sellal, F. 2022. “Anatomical and Neurophysiological Basis of Face Recognition.” Revue Neurologique 178, no. 7: 649–653. 10.1016/j.neurol.2021.11.002. 34863530

[ejn70014-bib-0044] Spunt, R. P. , and R. Adolphs . 2019. “The Neuroscience of Understanding the Emotions of Others.” Neuroscience Letters 693: 44–48. 10.1016/J.NEULET.2017.06.018. 28624265 PMC5732077

[ejn70014-bib-0045] Suzuki, A. , T. Hoshino , K. Shigemasu , and M. Kawamura . 2006. “Disgust‐Specific Impairment of Facial Expression Recognition in Parkinson's Disease.” Brain 129: 707–717. 10.1093/BRAIN/AWL011. 16415306

[ejn70014-bib-0046] Terruzzi, S. , G. Funghi , C. Meli , et al. 2023. “The FACE Test: A New Neuropsychological Task to Assess the Recognition of Complex Mental States From Faces.” Neurological Sciences 44, no. 7: 2339–2347. 10.1007/s10072-023-06697-w. 36849696 PMC10257594

[ejn70014-bib-0047] Tessitore, A. , A. R. Hariri , F. Fera , et al. 2002. “Dopamine Modulates the Response of the Human Amygdala: A Study in Parkinson's Disease.” Journal of Neuroscience 22, no. 20: 9099–9103. 12388617 10.1523/JNEUROSCI.22-20-09099.2002 PMC6757686

[ejn70014-bib-0048] Thye, M. , P. Hoffman , and D. Mirman . 2024. “The Neural Basis of Naturalistic Semantic and Social Cognition.” Scientific Reports 14, no. 1: 6796. 10.1038/s41598-024-56897-3. 38514738 PMC10957894

[ejn70014-bib-0049] Trompeta, C. , B. Fernández Rodríguez , and C. Gasca‐Salas . 2021. “What Do We Know About Theory of Mind Impairment in Parkinson's Disease?” Behavioral Sciences 11, no. 10: 130. 10.3390/bs11100130. 34677223 PMC8533307

[ejn70014-bib-0050] Wabnegger, A. , R. Ille , P. Schwingenschuh , et al. 2015. “Facial Emotion Recognition in Parkinson's Disease: An fMRI Investigation.” PLoS ONE 10, no. 8: e0136110. 10.1371/JOURNAL.PONE.0136110. 26285212 PMC4540566

[ejn70014-bib-0051] Wang, J. , L. Sun , L. Chen , et al. 2023. “Common and Distinct Roles of Amygdala Subregional Functional Connectivity in Non‐Motor Symptoms of Parkinson's Disease.” npj Parkinson's Disease 9, no. 1: 28. 10.1038/s41531-023-00469-1. PMC9938150 36806219

[ejn70014-bib-0052] Whitfield‐Gabrieli, S. , and A. Nieto‐Castanon . 2012. “Conn: A Functional Connectivity Toolbox for Correlated and Anticorrelated Brain Networks.” Brain Connectivity 2, no. 3: 125–141. 10.1089/brain.2012.0073. 22642651

[ejn70014-bib-0053] World Medical Association . 2013. “World Medical Association Declaration of Helsinki: Ethical Principles for Medical Research Involving Human Subjects.” JAMA 310, no. 20: 2191–2194. 10.1001/jama.2013.281053. 24141714

[ejn70014-bib-0054] Xia, M. , J. Wang , and Y. He . 2013. “BrainNet Viewer: A Network Visualization Tool for Human Brain Connectomics.” PLoS ONE 8, no. 7: e68910. 10.1371/journal.pone.0068910. 23861951 PMC3701683

[ejn70014-bib-0055] Yoshimura, N. , M. Kawamura , Y. Masaoka , and I. Homma . 2005. “The Amygdala of Patients With Parkinson's Disease Is Silent in Response to Fearful Facial Expressions.” Neuroscience 131, no. 2: 523–534. 10.1016/j.neuroscience.2004.09.054. 15708493

